# Dysfunction of mitochondria in intestinal epithelial cells: a key player in the pathogenesis of inflammatory bowel diseases

**DOI:** 10.1093/gastro/goag059

**Published:** 2026-06-19

**Authors:** Jiangmei Pang, Mingshan Jiang, Yongbin Jia, Lili Li, Yan Li, Hong Zhang, Yiding Chen, Kexin Chen, Hao Lin, Jingjing Chen, Jiaxin Li, Yongchun Shen, Zou Xiang, Hu Zhang

**Affiliations:** Department of Gastroenterology, West China Hospital, Sichuan University, Chengdu, Sichuan, P. R. China; Lab of Inflammatory Bowel Disease and Centre for Inflammatory Bowel Disease, West China Hospital, Sichuan University, Chengdu, Sichuan 610041, P. R. China; Department of Gastroenterology, West China Hospital, Sichuan University, Chengdu, Sichuan, P. R. China; Lab of Inflammatory Bowel Disease and Centre for Inflammatory Bowel Disease, West China Hospital, Sichuan University, Chengdu, Sichuan 610041, P. R. China; Lab of Inflammatory Bowel Disease and Centre for Inflammatory Bowel Disease, West China Hospital, Sichuan University, Chengdu, Sichuan 610041, P. R. China; Department of Gastroenterology, West China Hospital, Sichuan University, Chengdu, Sichuan, P. R. China; Lab of Inflammatory Bowel Disease and Centre for Inflammatory Bowel Disease, West China Hospital, Sichuan University, Chengdu, Sichuan 610041, P. R. China; Department of Gastroenterology, West China Hospital, Sichuan University, Chengdu, Sichuan, P. R. China; Lab of Inflammatory Bowel Disease and Centre for Inflammatory Bowel Disease, West China Hospital, Sichuan University, Chengdu, Sichuan 610041, P. R. China; Department of Gastroenterology, West China Hospital, Sichuan University, Chengdu, Sichuan, P. R. China; Lab of Inflammatory Bowel Disease and Centre for Inflammatory Bowel Disease, West China Hospital, Sichuan University, Chengdu, Sichuan 610041, P. R. China; Department of Gastroenterology, Affiliated Hospital of North Sichuan Medical College & Sichuan Branch of National Clinical Research Center for Digestive Diseases, Nanchong, Sichuan 637000, P. R. China; Department of Gastroenterology, West China Tianfu Hospital, Sichuan University, Chengdu, Sichuan 637000, P. R. China; Department of Gastroenterology, West China Hospital, Sichuan University, Chengdu, Sichuan, P. R. China; Lab of Inflammatory Bowel Disease and Centre for Inflammatory Bowel Disease, West China Hospital, Sichuan University, Chengdu, Sichuan 610041, P. R. China; Department of Gastroenterology, West China Hospital, Sichuan University, Chengdu, Sichuan, P. R. China; Lab of Inflammatory Bowel Disease and Centre for Inflammatory Bowel Disease, West China Hospital, Sichuan University, Chengdu, Sichuan 610041, P. R. China; Department of Gastroenterology, West China Hospital, Sichuan University, Chengdu, Sichuan, P. R. China; Lab of Inflammatory Bowel Disease and Centre for Inflammatory Bowel Disease, West China Hospital, Sichuan University, Chengdu, Sichuan 610041, P. R. China; Department of Gastroenterology, West China Hospital, Sichuan University, Chengdu, Sichuan, P. R. China; Lab of Inflammatory Bowel Disease and Centre for Inflammatory Bowel Disease, West China Hospital, Sichuan University, Chengdu, Sichuan 610041, P. R. China; Department of Respiratory and Critical Care Medicine, West China Hospital, West China School of Medicine, and Division of Pulmonary Diseases, State Key Laboratory of Biotherapy, Sichuan University, Chengdu, Sichuan 610041, P. R. China; Department of Health Technology and Informatics, Hong Kong Polytechnic University, Hong Kong SAR 999077, P. R. China; Department of Gastroenterology, West China Hospital, Sichuan University, Chengdu, Sichuan, P. R. China; Lab of Inflammatory Bowel Disease and Centre for Inflammatory Bowel Disease, West China Hospital, Sichuan University, Chengdu, Sichuan 610041, P. R. China

**Keywords:** inflammatory bowel diseases, mitochondria, intestinal epithelial cells, gut microbes, metabolism, crosstalk

## Abstract

Inflammatory bowel diseases (IBD) are a group of chronic gastrointestinal disorders with increasing incidence and prevalence that represent a major challenge for healthcare systems worldwide. Owing to their intricate and largely unknown etiologies, these patients are difficult to diagnose and treat. Intestinal barriers, including biological (gut microbes), chemical, and physical barriers, are crucial for maintaining normal intestinal function; thus, damage to intestinal barriers plays a role in the progression of intestinal inflammatory responses and hence in possible IBD. Intestinal epithelial cells (IECs), which contain mitochondria, play pivotal roles in intestinal barriers, which are indispensable for numerous functions, including energy production, signal transduction, cell-fate determination, epigenetic modifications, and inter-organelle crosstalk in cells. This review focuses on the current understanding of how mitochondria in IECs maintain and disturb intestinal-barrier homeostasis. We also discuss mitochondria-targeted therapeutics for IBD. These findings offer new insights into the etiological underpinnings of IBD, leading to the proposal of innovative therapeutic strategies. In the future, much remains to be done to understand the contribution of mitochondria in IECs to the functions of intestinal barriers.

## Graphical abstract

**Graphical Abstract.**
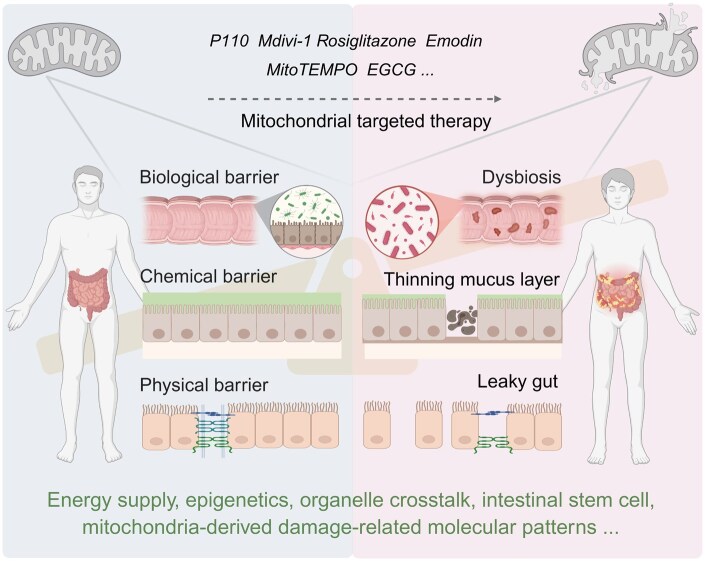
Created in BioRender. pang, J. (2026) https://BioRender.com/4zmnvm0

## Background

Inflammatory bowel diseases (IBD), mainly ulcerative colitis (UC) and Crohn’s disease (CD), represent a category of persistent gastrointestinal disorders characterized by alternating phases of spontaneous flare-ups and remission [1, 2], which are driven by complex interactions between genetic predispositions, environmental triggers, dysregulated immune responses, and alterations in the intestinal microbial composition [3, 4]. The prevalence of IBD is higher in Western developed countries and the global burden of IBD has demonstrated a sustained upwards trajectory, particularly in newly industrialized regions, where rapid shifts in environmental risk factors are considered primary drivers [5–8]. By 2021, the cumulative number of IBD cases worldwide had increased to 3.83 million, underscoring its emergence as a significant public health challenge [9, 10]. Currently, first-line therapies include aminosalicylates, corticosteroids, and immunosuppressants, with an array of new therapies including biologics and small-molecule drugs [11–15]. While reducing severe symptoms, these treatments remain costly [16] and inconsistently effective, with only 20%–30% remission rates [17, 18], and increase the risk of infection through immunosuppression [19–21]. Additionally, lifelong disease management is burdened by relapse risks, surgical interventions, and comorbidities such as colorectal cancer [22–24]. These unmet clinical needs underscore the urgency of exploring novel pathogenic mechanisms and therapeutic targets.

The intestinal-barrier function is achieved through the integrated operation of three distinct but cooperative lines of defense: (i) physical defense: served by the mucus layer (a MUC2-based gel) as the first-line physical sieve and the intestinal epithelium with its tight junctions as the second cellular wall; (ii) chemical defense: mediated by a suite of soluble antimicrobial factors, including peptides (e.g. defensins and lysozyme) secreted by Paneth cells (PCs) [25], trefoil factor 3 secreted by goblet cells, enzymes, and inorganic ions, which are secreted into the mucus and lumen to chemically neutralize threats; (iii) biological defense: provided by the resident commensal microbiota through the competitive exclusion of pathogens, reinforcement of the host’s physical and chemical barriers, and precise modulation of mucosal immunity, and the mucosal immune system, together forming a sophisticated biological shield [26, 27]. In healthy states, intestinal epithelial cells (IECs) dynamically regulate intestinal-barrier integrity through energy-dependent processes, including mucus secretion by goblet cells, tight-junction formation, and continuous epithelial renewal by intestinal stem cells (ISCs), which are first compromised before the onset of IBD, leading to a “leaky gut” [28]. In addition to sustaining energy supply for the integrity of the intestinal barriers, the mitochondria in IECs accomplish this task through many other mechanisms. Therefore, emerging evidence implicates mitochondrial dysfunction in IECs as a fundamental driver of barrier failure, linking metabolic stress to immune activation.

The mitochondria in IECs are not merely adenosine triphosphate (ATP) producers, but also multifunctional hubs that govern redox balance, metabolic signaling [29], and inter-organelle crosstalk [30, 31], including the modulation of nuclear epigenetics through metabolites such as acetyl-CoA and α-ketoglutarate [32]. They also sustain high-energy demands for epithelial turnover, nutrient absorption, and the complex assembly of tight junctions. However, in IBD, stressors such as microbial metabolites or genetic variants disrupt mitochondrial networks, triggering bioenergetic collapse, reactive oxygen species (ROS) overproduction, and mitochondrial DNA (mtDNA) leakage. These defects impair barrier-repair mechanisms and amplify inflammatory cascades, positioning mitochondrial dysfunction as a nexus between epithelial injury and immune hyperactivity. Emerging evidence implicates mitochondrial dysfunction in IECs as a pivotal driver of IBD pathogenesis, although causal mechanisms remain incompletely defined.

While several excellent reviews have discussed the general role of mitochondria in IBD, most have focused on immune-cell metabolism or broad oxidative stress. This review distinguishes itself by specifically focusing on IECs as a central hub, integrating emerging concepts such as mitochondria–organelle interactions, epigenetic reprogramming, and the mitochondria–microbiota feedback loop. We provide a novel framework that positions mitochondrial dysfunction not just as a consequence of inflammation, but as a primary driver of barrier failure and disease chronicity.

## Mitochondrial structure and function in IECs

Mitochondria govern intestinal epithelial homeostasis through a coordinated suite of dynamic processes. Their roles form a functional continuum: from internal quality control, to serving as the central energy and metabolic hub, to acting as a platform for inter-organellar communication, and ultimately mediating systemic dialogue with the gut microbiota and immune system. The following sections detail these interconnected mechanisms, beginning with the foundational systems that maintain the mitochondrial network itself.

### Mitochondrial structure and dynamics

**Figure 1:**
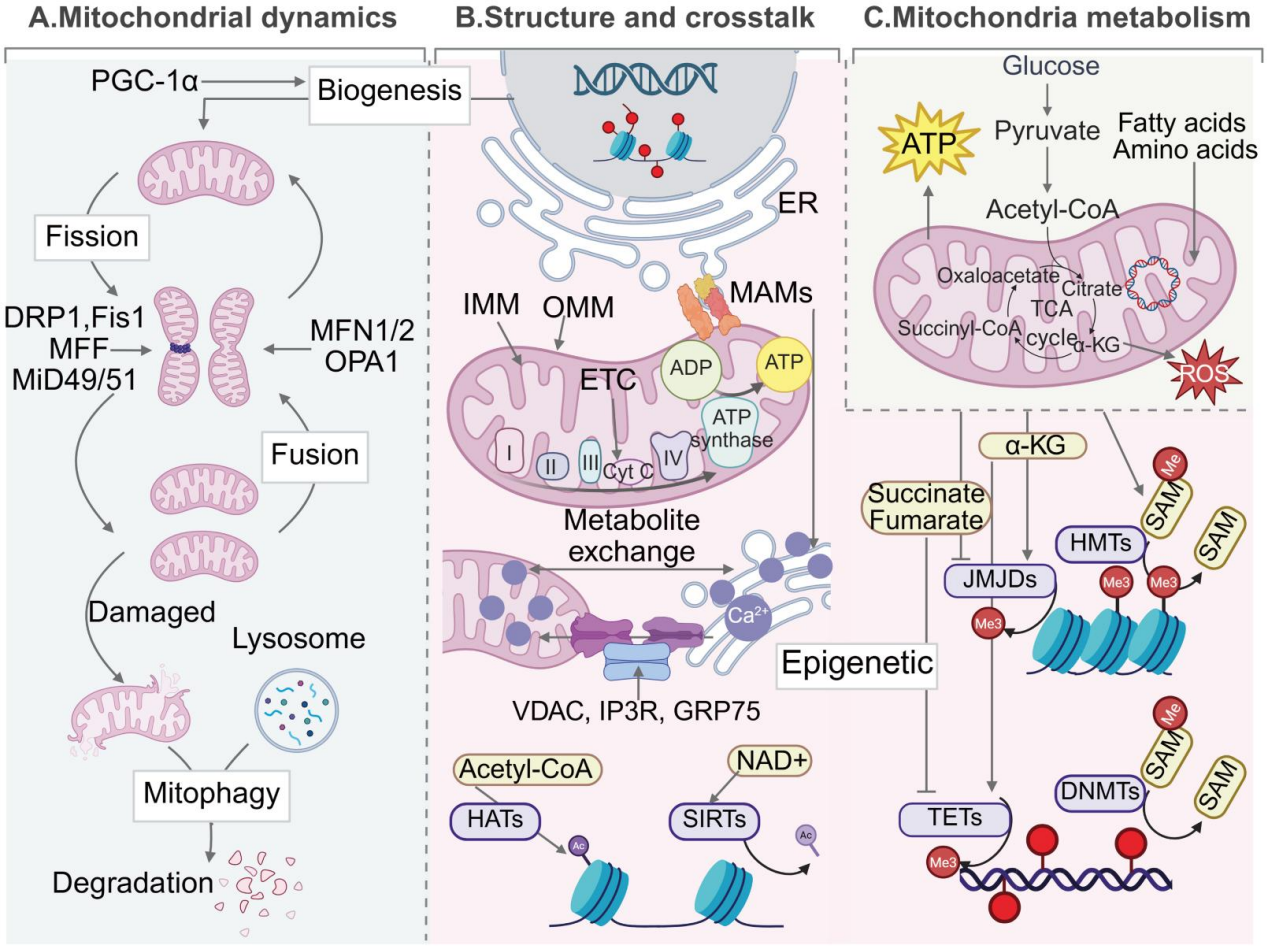
Normal functions and structure of mitochondria. (A) Mitochondrial dynamics and quality control. Mitochondrial homeostasis is maintained by balanced fission [dynamin-related protein 1 (DRP1), fission 1 (Fis1), mitochondrial fission factor, mitochosndrial dynamics proteins of 49/51 kDa (MiD49/51)] and fusion [mitofusin 1/2 (MFN1/2), optic atrophy 1 (OPA1)], as well as peroxisome-proliferator-activated receptor gamma coactivator 1-alpha (PGC-1α)-driven biogenesis and mitophagic clearance of damaged mitochondria. (B) Mitochondrial structure and inter-organelle crosstalk. Mitochondria are double-membraned organelles with an outer mitochondrial membrane and an inner mitochondrial membrane (IMM), with the latter housing the electron transport chain (ETC) for oxidative phosphorylation. Mitochondria-associated membranes (MAMs) mediate material exchange between mitochondria and the endoplasmic reticulum (ER). Mitochondrial metabolites, including acetyl-CoA, α-ketoglutarate (α-KG), S-adenosyl methionine, and nicotinamide adenine dinucleotide (NAD^+^), translocate into the nucleus, acting as substrates or cofactors for epigenetic enzymes: histone acetyltransferases (HATs), histone methyltransferases (HMTs), Jumonji domain-containing demethylases (JMJDs), DNA methyltransferases (DNMTs), ten-eleven translocation dioxygenases, and sirtuins (SIRTs). This process represents mitochondria–nuclear crosstalk that regulates nuclear epigenetic modifications. (C) The tricarboxylic acid (TCA) cycle sustains mitochondrial amino acid and fatty acid metabolism, supporting adenosine triphosphate (ATP) production and ROS generation. Created in BioRender. pang, J. (2026) https://BioRender.com/nn4eu77.

Mitochondria are semiautonomous organelles with circular mtDNA (∼16.6 kb) [33]. Mitochondrial fission, mediated by dynamin-related protein 1 (DRP1) and regulated by mitochondrial fission 1 protein (Fis1), mitochondrial fission factor, and mitochondrial dynamics proteins of 49/51 kDa, is balanced against fusion driven by mitofusin 1/2 and optic atrophy 1 (OPA1). These mitochondrial dynamics regulate mitochondrial distribution, metabolic flexibility, and damage segregation [34, 35]. Damaged mitochondria are subsequently cleared via ubiquitin-dependent (PINK1/Parkin) or receptor-mediated (BNIP3L/NIX, FUNDC1, FKBP8, etc.) pathways referred to as mitophagy [36–38], whereas peroxisome proliferator-activated receptor gamma (PPARγ) coactivator 1-α (PGC-1α) coordinates biogenesis by activating the NRF1/2–ERRα–mtTFA axis to replenish mtDNA and mitochondrial proteins [39]. The efficiency of fission and fusion, as well as that of mitophagy and biogenesis, must be balanced to maintain the number of mitochondria in each cell (Fig. 1a). Further integrating stress adaptation, the mitochondrial unfolded protein response deploys chaperones [e.g. heat shock protein (HSP) 60] to resolve proteotoxicity and fine-tunes the tricarboxylic acid (TCA) cycle and oxidative phosphorylation (OXPHOS) flux—functions extending beyond its original role in mtDNA maintenance [40, 41]. Together, these spatially coordinated processes constitute a dynamic network that is aimed at keeping mitochondria in order to sustain energy production, redox equilibrium, and organelle renewal.

### Energy production, ROS regulation, and metabolic signaling

Mitochondria execute the final and most efficient stages of cellular energy harvest through a coordinated sequence of reactions. Carbohydrate-derived pyruvate is transported into the matrix and decarboxylated to acetyl-CoA by the pyruvate dehydrogenase complex. Acetyl-CoA then enters the TCA cycle—a central metabolic hub. Within this cycle, a series of oxidation steps—catalysed by key enzymes such as isocitrate dehydrogenase and α-ketoglutarate dehydrogenase—completely oxidizes acetyl-CoA, generating CO_2_, GTP, and the crucial reducing equivalents NADH and FADH_2_ (from succinate dehydrogenase). This cycle also interconnects with other catabolic pathways: fatty acid β-oxidation (FAO) recurrently cleaves fatty acids into acetyl-CoA units, concurrently producing NADH and FADH_2_; similarly, amino acid carbon skeletons, after deamination, are converted into various TCA cycle intermediates (e.g. α-ketoglutarate, oxaloacetate) for oxidation [42].

The reducing power captured in NADH and FADH_2_ is subsequently utilized in OXPHOS. These molecules donate electrons to the electron transport chain (ETC)—comprising complexes I–IV and mobile carriers [ubiquinone, cytochrome c (Cyt c)]—embedded in the inner mitochondrial membrane (Fig. 1b) [43]. The exergonic flow of electrons through complexes I, III, and IV drives the active translocation of protons across the membrane, establishing an indispensable electrochemical proton gradient. This stored potential energy is finally converted into chemical energy as protons flow back into the matrix through ATP synthase (Complex V), driving the phosphorylation of ADP to ATP [44]. Given its role as the integrative core of cellular energetics, disruption of any component within this sequential pathway—from substrate oxidation to proton coupling—compromises ATP production and metabolic homeostasis. It is therefore consequential that mitochondrial dysfunction is a recognized contributor to the pathogenesis of IBD, linking bioenergetic failure to epithelial-barrier defects and sustained inflammation [45, 46].

Notably, OXPHOS is imperfect—0.2%–2% of electrons that leak prematurely react with oxygen to form ROS [47]. While physiological ROS act as signaling molecules, modulating processes such as differentiation, apoptosis, and immune responses [48], their overproduction overwhelms antioxidant defense systems such as superoxide dismutase and glutathione peroxidase [49], leading to oxidative damage. This delicate balance underscores mitochondria as both ROS sources and sentinels of cellular stress adaptation. ROS are also conducive to maintaining intestinal epithelial homeostasis because most apical IECs produce more ROS than their antioxidant capacity, which fosters a proapoptotic fate [50]. Unfortunately, excessive accumulation of ROS can disrupt intestinal barriers and activate inflammatory cascades, resulting in intestinal inflammation.

### Inter-organellar crosstalk

In addition to functioning relatively independently, mitochondria also establish extensive physical and functional interactions with other cellular organelles through membrane contact sites. Mitochondria-associated endoplasmic reticulum (ER) membranes (MAMs), tethered by proteins such as mitofusin 1/2, VDAC, IP3R, and GRP75, regulate calcium homeostasis, lipid metabolism, and mitochondrial dynamics (fusion/fission) while coordinating stress responses and autophagy [51, 52] (Fig. 1b). Mitochondria–lysosome interactions, which are classically mediated by Rab7 GTPase [53], facilitate metabolite exchange (e.g. calcium, iron, cholesterol) and mitophagy to eliminate damaged mitochondria [54, 55]. In addition, emerging evidence highlights mitochondrial crosstalk with the Golgi, peroxisomes, lipid droplets [56], etc., underscoring the integrative role of mitochondria in cellular metabolism and homeostasis. Collectively, the intricate web of inter-organelle crosstalk serves as a cellular metabolic control center for intestinal epithelium homeostasis.

However, compared with the aforementioned pathways, mitochondria–nucleus crosstalk, which is essential for cellular homeostasis, operates through distinct mechanisms (Fig. 1c). Notably, most mitochondrial proteins are encoded by the nucleus. Conversely, intermediates of mitochondrial metabolism can serve as substrates for epigenetic modifications, thereby regulating nuclear gene expression. Histone acetylation catalysed by histone acetyltransferases that obtain an acetyl group from acetyl-CoA is associated with chromatin relaxation and transcriptional activation. Conversely, intracellular nicotinamide adenine dinucleotide (NAD)^+^ levels regulate the activity of class III histone deacetylases (sirtuins) [32]. In a manner dependent on S-adenosylmethionine, histone methylation catalysed by histone methyltransferases regulates gene transcription and DNA methylation—catalysed and maintained by DNA methyltransferase 1 (DNMT1)—inhibits gene expression and both can be demethylated by α-ketoglutarate, a key co-substrate for the JMJD ten-eleven translocation family of dioxygenases. The demethylation process can be inhibited by succinate and fumarate [32]. Epigenetics assists the normal function of IECs at a physiological level. Elevated α-ketoglutarate, arising either from aged mitochondria in an ISC subpopulation to promote niche renewal or from lineage-specific remodeling of the TCA cycle to promote repair in colitis, converges on the activation of ten-eleven translocation dioxygenases that drive DNA demethylation and epigenetic reprogramming that is critical for secretory cell-fate determination [57, 58]. In other words, the mitochondrial regulation of intestinal physiology extends beyond metabolic support to direct nuclear reprogramming, suggesting transcriptional control over intestinal homeostasis.

## Mitochondrial regulation of intestinal-barrier homeostasis

### The mitochondria–microbiota interaction maintains the biological barrier

The gastrointestinal tract harbors a dense microbial ecosystem (> 10^14^ microorganisms, > 2,000 species), which is mostly located in the colon and profoundly shaped by mitochondrial activity in IECs [59–62]. Mitochondrial FAO and OXPHOS deplete luminal oxygen, creating a hypoxic niche that favors healthy obligate anaerobes dominated by *Firmicutes* that produce short-chain fatty acids (SCFAs) to fuel the mitochondrial OXPHOS of IECs, thus forming a positive feedback loop [29, 63]. Conversely, “physiologic hypoxia” is capable of stabilizing hypoxia-inducible factor (HIF), which in turn triggers the expression of HIF-regulated barrier-protective genes, modulates epithelial metabolic processes, and maintains mucosal homeostasis [64].

The HIF complex, formed by one of the three α subunits (HIF-1α, -2α, -3α) and a constitutive β subunit, acts as a master oxygen sensor with its activity controlled by oxygen levels through prolyl hydroxylase domain (PHD)-mediated degradation and factor-inhibiting HIF-1-mediated inactivation of the α subunits, respectively. Their function in the gut demonstrates cell-type/isoform-specific and segment-specific (Section “the mitochondria function help maintain ISCs function”) duality, leading to both protective and pathogenic outcomes. In IECs, HIF-1α predominantly exerts a barrier-protective role. It enhances mucosal defense by regulating the secretion of antimicrobial peptides (AMPs), mucins, and trefoil factor 3, and by supporting tight-junction integrity [65]. It promotes post-injury healing [66] and protects IECs from ferroptosis [67, 68]. HIF-1α deficiency exacerbates intestinal-barrier dysfunction in mice [69]. Conversely, HIF-2α activation in IECs is largely pathogenic, driving inflammation and disrupting the barrier. This isoform-specific dichotomy extends to immune cells within the mucosal environment. For instance, HIF-1α in B cells is essential for IgA-producing cell differentiation and its loss exacerbates colitis [70]. In CD4+ T cells, HIF-2α, but not HIF-1α, induces miR-29a to attenuate TH1-mediated colitis [71]. Furthermore, mesenchymal stem cells overexpressing HIF-1α can ameliorate colitis by modulating macrophage polarization [72]. The therapeutic potential of targeting this pathway is underscored by the protective effect of epithelial PHD inhibition in experimental colitis, which stabilizes HIF and enhances barrier function [73]. Promisingly, a gut-restricted PHD 1/2 inhibitor, ISM012-042, has shown efficacy in restoring barrier integrity and reducing inflammation in preclinical models [74]. Furthermore, clinical trials have been conducted with the HIF-1α stabilizer GB004 and the PHD inhibitor AKB-4924 (Aerpio Therapeutics) for IBD treatment [75, 76]. Also, knockout of the factor-inhibiting HIF-1 in colon epithelium attenuates chronic colitis in experimental mice [77]. In summary, the HIF pathway, modulated by mitochondrial and tissue oxygen dynamics, serves as a central regulator of intestinal homeostasis. Its complex, cell-specific roles—from directly fortifying the epithelial barrier to finely tuning mucosal immunity—make it a compelling therapeutic target for barrier-dysfunction diseases such as IBD. These findings indicate that mitochondria in IECs help maintain the homeostasis of intestinal microbes, thereby contributing to the maintenance of the intestinal biological barrier.

**Figure 2:**
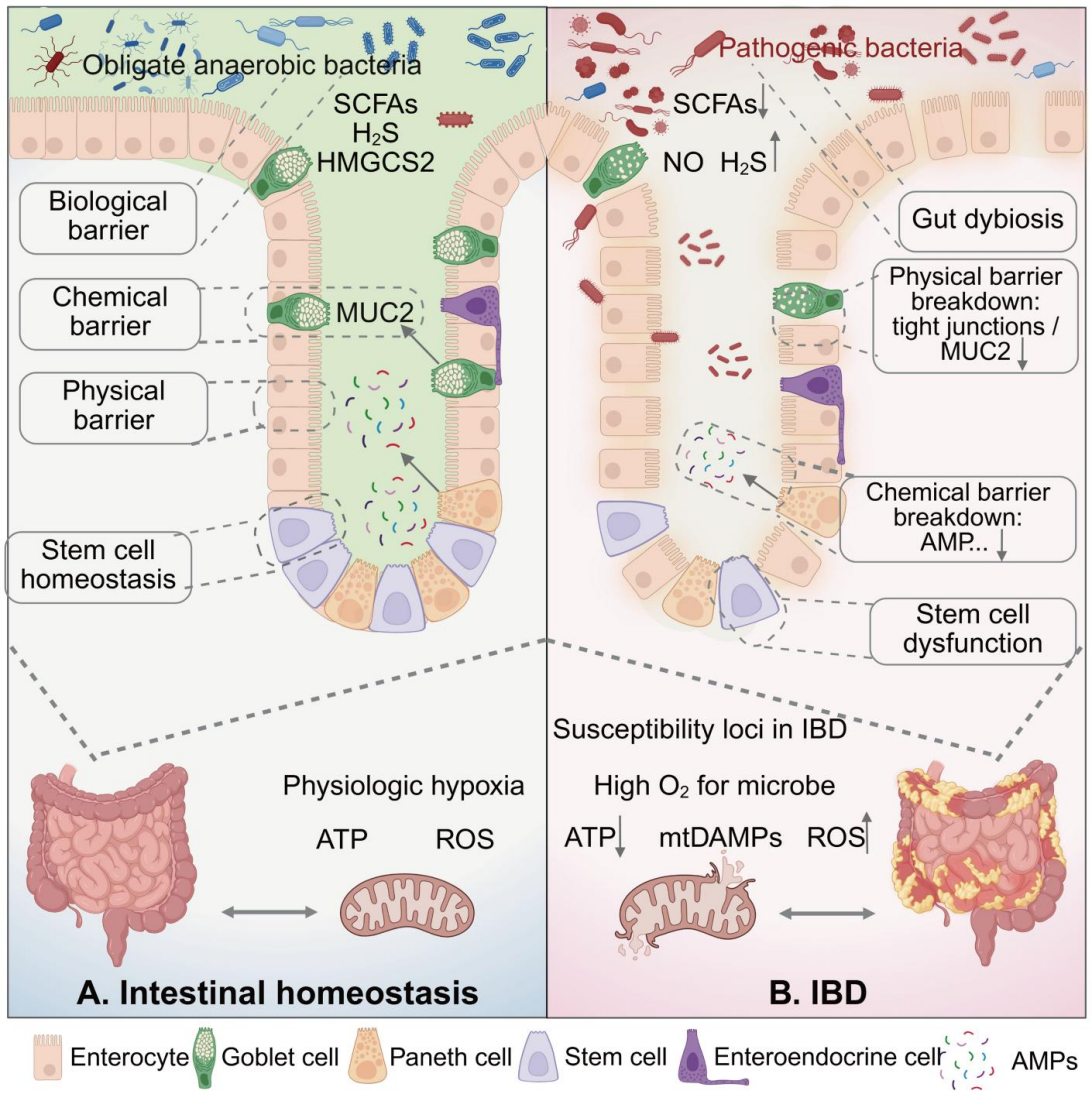
Mitochondria in IECs from intestinal homeostasis to IBD. (A) Under homeostatic conditions, IEC mitochondria maintain intestinal-barrier integrity by sustaining luminal physiological hypoxia, which supports obligate anaerobic bacteria producing beneficial metabolites such as SCFAs and 3-hydroxy-3-methylglutaryl-CoA synthase 2 (HMGCS2). Mitochondria also provide adequate adenosine triphosphate (ATP) and moderate ROS to support barrier function and stem-cell homeostasis. (B) In IBD, IEC mitochondrial dysfunction disrupts luminal hypoxia, driving gut dysbiosis with the expansion of pathogenic bacteria. These pathogens further impair barrier function by reducing SCFAs, altering nitric oxide (NO) and hydrogen sulfide (H_2_S) levels, and exacerbating the breakdown of physical [tight junctions, mucin 2 (MUC2)] and chemical (AMPs) barriers. Concurrently, impaired ATP production, excessive ROS, and the release of mitochondrial-damage-associated molecular patterns (mtDAMPs) amplify inflammation, leading to “leaky gut” and promoting IBD pathogenesis. Created in BioRender. pang, J. (2026) https://BioRender.com/mjyalx1.

Microbiota-derived metabolites, particularly SCFAs, engage in bidirectional crosstalk with mitochondria. SCFAs affect mitochondrial energy metabolism by binding free fatty acid receptors 2/3 to increase mitochondrial FAO [78]. While sodium butyrate directly upregulates mitochondrial respiration and bioenergetic genes (e.g. TFAM, NDUFA1/4/6, COX6A1, ATP5E/8) [79], succinate supplementation rescues mitochondrial dysfunction in PHB1-deficient epithelium, mitigating enteritis by restoring TCA cycle flux [80]. Physiological levels of hydrogen sulfide are oxidized by sulfide quinone oxidoreductase at the CoQ site, which donates electrons to fuel OXPHOS [81]. Collectively, metabolites from intestinal microbes can act to fuel the mitochondrial energy production process. Moreover, Bass *et al.* reported that 3-hydroxy-3-methylglutaryl-CoA synthase-2 (HMGCS2), which serves as a key rate-limiting enzyme in the ketogenesis pathway, is expressed in the large intestine and is markedly downregulated in CD and UC patients [82]. However, colonic bacterial metabolites can increase HMGCS2 expression in human colon epithelial cell lines [82] and studies have revealed associations between HMGCS2 and Wnt/β-catenin/PPAR-γ signaling and its function in regulating IEC differentiation [83, 84], but whether intestinal microbes are involved needs further study. This evidence suggests that intestinal microbes can regulate mitochondrial ketogenesis. In conclusion, the bidirectional interaction between mitochondria and the microbiota contributes to biological barrier sustenance, and hence is beneficial to intestinal health (Fig. 2a).

### Mitochondria maintain the integrity of chemical and physical barriers

The chemical and physical intestinal barriers also rely heavily on the proper function of IECs and their mitochondria. Mitochondria underpin the chemical barrier through a dual role: they are essential for the biosynthesis and secretion of its effector molecules and they concurrently maintain the health and function of the secretory cells (e.g. PCs) themselves [85]. The mucus layer—a key physical barrier component primarily composed of MUC2—depends on mitochondrial support. Notably, mitochondria not only provide the energy required for the synthesis, transport, packaging, and secretion of the MUC2 protein, but also play a pivotal role in the differentiation of ISCs into goblet cells through Forkhead Box O and Notch signaling-regulated mitochondrial fission [86]. Mitochondrial dysfunction decreases the amount of MUC2 protein [87]. Furthermore, at the core of the cellular physical barrier are the tight junctions between adjacent IECs, including occludin, claudins, and zonula occludens [88]. A recently published article revealed that, by affecting mitochondrial functions, banana starch nanoparticles can reduce the integrity of tight junctions [89]. Mitochondria-associated functions, including energy supplementation, ROS and Ca2+ homeostasis modulation, and interactions with intestinal microbes, are vital for the production and maintenance of tight junctions. In brief, mitochondria in IECs are essential for the integrity of intestinal chemical and physical barriers, thereby building a “wall” against microbial migration, mechanical damage, etc. (Fig. 2a).

### The mitochondria in IECs help maintain ISCs function

Despite the protection of biological and chemical barriers, IECs are exposed to mechanical abrasion, extreme pH variations, and harmful bacteria to a great extent due to their enlarged surface, which is amenable to nutrient absorption [90, 91]. Therefore, these IECs must renew quickly to prevent destruction via balanced self-renewal and the differentiation of ISCs relies on mitochondrial activity. Pathological phenotypes of ISC function caused by disrupted mitochondria have been observed; for example, scientists have reported that genetic deletion of HSP60 causes a loss of ISC stemness and a decrease in ISC numbers [85].

Mitochondrial energy metabolism can influence the cell fate of ISCs through several pathways. Taking energy supply as an example, ISCs with a defective ETC exhibit impaired proliferation and enteroblast production, ultimately failing to generate mature IECs [92]. Compared with adjacent PCs, ISCs have more mitochondria with increased activity, which provide sufficient energy for the rapid renewal of IECs. This also leads to a subsequent increase in ROS, promoting the activation of p38, which drives changes from immature ISCs to mature ISCs and PCs. Concurrently, PCs exhibit more active glycolysis, thereby providing lactate to ISCs as a substrate for OXPHOS [93]. Emerging evidence has revealed that mitochondrial FAO also plays a role in maintaining ISC function. Deletion of Cpt1a (the rate-limiting enzyme in FAO) [94, 95], double knockout of HNF4A/G transcription factors, which directly activate FAO genes [96], and deletion of PRDM16, which transcriptionally controls FAO in crypts [97], lead to a loss of ISC numbers and functions. These studies suggest that mitochondria supply energy to ISCs for their proliferation and differentiation. In addition, ISC functions are regulated by pyruvate and ketone body metabolism [98]. Mitochondrial dynamics can also modulate ISCs; for example, mitochondrial fission is necessary for ISC differentiation into PCs and goblet cells through the Notch/Forkhead Box O/miR–484/Fis1 axis [86]. Notably, in YAP+ ISCs―which may be an earlier intestinal cell origin that determines Lgr5+ cell production―the mitochondrial molecule ECSIT regulates YAP protein translation [99].

Beyond these direct metabolic roles, mitochondrial oxygen consumption also significantly shapes the stem-cell niche by modulating HIF signaling, the impact of which on ISCs exhibits a striking segment-specific duality. In the ileum, hypoxia and HIF-1α stabilization drive a metabolic shift toward glycolysis that compromises ISC function and regenerative capacity [100]. Similarly, hypoxia exerts a significant negative impact on the formation of jejunum-derived organoids [101]. In stark contrast, human colon-derived organoids show no significant difference in formation efficiency between normoxia and hypoxia (2% O_2_) [102]. This resilience aligns with the physiology of the distal colon crypt, in which stabilized HIF-1α integrates into an adaptive circuit that cooperates with Wnt signaling to metabolically promote ISC self-renewal and barrier maintenance [103]. This fundamental difference highlights the way in which ISCs have evolutionarily adapted their HIF-mediated responses to the distinct physiological oxygen gradients along the gut axis. In summary, mitochondria play a role in ISC stemness maintenance or differentiation fate determination through many mechanisms (Fig. 2a) and many unknown mechanisms are worth exploring.

## Mitochondrial dysfunction in IBD pathogenesis

In general, before the onset of IBD, the intestinal epithelial barriers are first compromised [104]. When the first line of defense is breached, microbial antigens in the gut lumen will translocate into the intestinal mucous. In the second phase, immune cells in the intestinal mucous mount a strong immune response against the translocated antigens, resulting in the production of many cytokines (such as CCL2, IL-12, IL-21, IL-2, TNF, and IFN-γ), leading to inflammatory reactions in the mucosa [105, 106] (Fig. 2b). A growing number of studies are trying to elucidate the pathological mechanisms of IBD, which are interconnected with each other and caused by impaired mitochondria in IECs. For example, impaired OXPHOS leads to excessive ROS that ruins intestinal barriers. However, to systematically describe these mechanisms more conveniently, we introduce them separately below.

### Primary mitochondrial defects

#### Bioenergetic failure results in an inadequate energy supply for IECs

IBD are characterized by impaired mitochondrial functions in IECs, which underpin epithelial-barrier dysfunction and perpetuates chronic inflammation. The physiological functions of mitochondria mentioned above are based on their normal morphology, which can be disturbed by numerous factors, resulting in significant alterations in IBD, including swelling and crista destruction [107–110]. Positioning morphology as fundamental for all functions, there is no surprise that the destruction of mitochondrial morphology results in energy defects, thus disturbing the normal functions of IECs.

A recent study demonstrated that the TCA cycle is suppressed in IBD patients [111]. Moreover, OXPHOS is the main way in which IECs produce energy and can be disrupted not only by morphological abnormalities, but also by decreased levels of ETC complexes, which exhibit both clinical and experimental consistency, although with notable inter-study variations. Clinical evidence has demonstrated decreased complex I expression in UC cohorts [112, 113]. Parallel investigations in experimental models reinforce these findings, with dextran sulfate sodium (DSS)-induced colitis mice exhibiting significant suppression of ETC-complex activity. Furthermore, sex-specific pathological patterns have emerged in IBD animal models, with female mice showing concurrent reductions in complex I and IV activities [114]. These discrepancies likely arise from individual differences, methodological divergences, and the complexity of the ETC itself. These changes hinder metabolic functions, especially OXPHOS, disrupt epithelial barriers, and promote microbial imbalance, exacerbating intestinal inflammation and disease progression.

Mitochondrial dynamics are also involved. A study conducted by Mancini *et al.* demonstrated that DSS-induced colitis mice presented increased mRNA expression of mitochondrial fission (i.e. DRP1 and Fis1)- and fusion (OPA1)-related molecules, as well as phospho-DRP1 [115]. Reduced expression of the OPA1 gene is also observed in IBD patients [116]. However, microarray analysis of data from a DSS-induced colitis dataset (GSE76658) revealed that DRP1-induced fission but not DRP1 fusion was upregulated [117]. Mucosal repair is suppressed by excessive mitochondrial fission via impairing butyrate metabolism in IECs [118] and increases the level of ROS, which promote IEC PANoptosis [117]. Suppression of mitochondrial fission through the delivery of P110 and metformin can reduce the severity of colitis [115, 119]. However, the promotion of mitochondrial fusion by vitamin A can prevent excessive inflammation [109]. Mitophagy eliminates dysfunctional mitochondria, the absence of which results in anomalously activated inflammation in DSS-induced colitis and IBD patients [120], and the relationship between mutations in mitophagy-related genes and IBD will be described in section “Mitochondria-function-related genes in IECs are also susceptibility genes for IBD”. In total, through the inhibition of mitochondrial fission and the promotion of mitochondrial fusion and enhancement of mitophagy, experiments have shown that an exosome can alleviate UC [121]. However, intriguingly, scientists have reported that Parkin exacerbates colitis by promoting vitamin D receptor degradation via the p62-mediated autophagy–lysosome pathway. Conversely, Parkin deficiency reduces colitis susceptibility and enhances resistance to DSS-induced colitis [122]. This observation suggests that overactivated mitophagy may exert paradoxical detrimental effects. Given the intricate relationship between mitochondrial dynamics and intestinal inflammation, further research is expected to elucidate the precise mechanisms involved, facilitating the development of mitochondrial dynamics-targeted therapeutic strategies for IBD.

Emerging evidence implicates impaired mitochondrial biogenesis in IBD pathogenesis. Studies have confirmed that the expression of PGC-1α—a key regulator of mitochondrial biogenesis—is significantly reduced in the colonic tissues of IBD patients. This downregulation is observed in both UC and CD, with the decreases in *PPARG* and *PPARGC1A* being more pronounced in CD than in UC [112, 123]. Mechanistically, Hou *et al.* reported SMYD5 upregulation and concurrent PGC1α suppression in IBD patients and murine models, where SMYD5 ablation protected against colitis by preserving epithelial mitochondrial function [124]. Clinically, reduced PGC1α has emerged as a diagnostic biomarker in UC [112]. While Novak *et al.* proposed NAD^+^ depletion-mediated SIRT1 inactivation as a mechanism impairing mitochondrial biogenesis [125], the precise regulatory pathways governing PGC1α downregulation remain undefined. In summary, downregulated mitochondrial biogenesis contributes to IBD pathogenesis, so the targeted reactivation of PGC1α represents a promising therapeutic strategy to restore mitochondrial homeostasis and augment current IBD treatments.

#### Mitochondrial-function-related genes in IECs are also susceptibility genes for IBD

Genetic predisposition is one of the etiologies of IBD, with ∼5% of the IBD susceptibility genes identified from human genome-wide association studies involved in mitochondrial homeostasis [126]. Examples include solute carrier family 25 member 11 (*SLC25A11*), encoding an oxoglutarate carrier that is essential for energy metabolism and redox balance [127, 128], and research has shown that increased SLC25A11 expression is positively associated with UC risk [129]. Ring finger protein 186 (*RNF*186) encodes an E3 ubiquitin ligase and *RNF186*^–/–^ mice suffer from severe acute colitis after DSS treatment [130]. Moreover, *SLC22A4* and *SLC22A5* are associated with the development of UC [131, 132]. These genes encode organic cation transporters novel 1/2, which work to transport several compounds, including carnitine, acetylcholine, and gut microbiota byproducts [133]. Moreover, the *HSPA1L* genes encoding HSP70 are also associated with IBD [134, 135]. Mitophagy-related genes, including the autophagy-related 16 like 1 gene (*ATG16L1*) [136, 137], calcium binding and coiled-coil domain 2 (*CALCOCO2*) [138], peroxisomal biogenesis factor 13 (*PEX13*), and SMAD-specific E3 ubiquitin protein ligase 1 (*SMURF1*) [139], are also associated with IBD. However, the clustering of mitochondrial-function-related genes in IBD susceptibility are not IEC-specific. The mitochondrial-function-related genes associated with IBD mentioned in this section are summarized in Table 1.

**Table 1:** Mitochondrial-function-related genes as susceptibility loci in IBD.

Mitochondria-function-related genes	Role of corresponding protein	References revealing correlations between mitochondrial-function-related genes and IBD
*SLC25A11*	Oxoglutarate carrier [127, 128]	[129]
*RNF186*	E3 ubiquitin ligase [130]	[130]
*SLC22A4/5*	Transporters for several compounds [133]	[131, 132]
*HSPA1L*	Encode HSP 70 involved in the UPR [134, 135]	[134, 135]
*ATG16L1*	Mitophagy [136]	[137]
*CALCOCO2*	Mitophagy [138]	[138]
*PEX13*	Mitophagy [139]	[139]
*SMURF1*	Mitophagy [139]	[139]

*SLC25A11* = solute carrier family 25 member 11, *RNF186* = ring finger protein 186, *SLC22A4/5* = solute carrier family 22 member 4/5, *HSPA1L* = heat shock protein family A member 1 like, UPR = unfolded protein response, *ATG16L1* = autophagy-related 16 like 1, *CALCOCO2* = calcium binding and coiled-coil domain 2, *PEX13* = peroxisomal biogenesis factor 13, *SMURF1* = SMAD-specific E3 ubiquitin protein ligase 1.

#### Organelle crosstalk disruptions in IECs are associated with the onset and disease features of IBD

**Figure 3:**
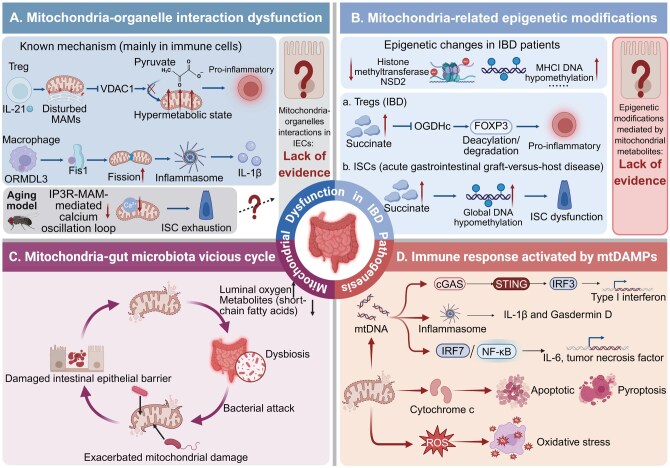
Detailed mechanisms linking mitochondrial dysfunction to IBD pathogenesis. (A) Mitochondria–organelle interaction dysfunction. Dysregulated interactions between mitochondria and other organelles have been reported in immune cells in IBD, but direct evidence for such mechanisms in IECs remains lacking. (B) Mitochondria-related epigenetic modifications. Altered mitochondria-associated epigenetic changes have been identified in IBD, including in regulatory T (Treg) cells and ISCs in the context of acute gastrointestinal graft-versus-host disease. However, direct evidence for IEC mitochondrial-mediated epigenetic modifications in IBD remains lacking. (C) Mitochondria–gut microbiota vicious cycle. Impaired mitochondrial function elevates luminal oxygen levels and reduces beneficial metabolites (e.g. SCFAs), driving gut dysbiosis. In turn, pathogenic bacteria attack the damaged intestinal epithelial barrier, exacerbating mitochondrial injury and further disrupting barrier integrity, creating a self-reinforcing dysfunctional loop. (D) Immune response activated by mitochondrial-damage-associated molecular patterns (mtDAMPs). Released mitochondrial DNA (mtDNA) engages three key inflammatory signaling axes: the cyclic GMP–AMP synthase (cGAS)–stimulator of interferon genes (STING) pathway, the inflammasome, and interferon regulatory factor 7 (IRF7)/nuclear factor-κB (NF-κB) signaling. Cytochrome c drives apoptosis, while ROS induces oxidative stress, collectively amplifying intestinal-tissue injury. IL-21 = interleukin-21, MAMs = mitochondria-associated membranes, VDAC1 = voltage-dependent anion channel 1, ORMDL3 = orosomucoid-like 3, Fis1 = fission 1, NLRP3 = NOD-like receptor family pyrin domain-containing 3 inflammasome, IL-1β = interleukin-1β, IP3R = inositol 1,4,5-trisphosphate receptor, NSD2 = nuclear receptor binding SET domain protein 2, MHCI = major histocompatibility complex class I, OGDHc = oxoglutarate dehydrogenase complex, FOXP3 = Forkhead Box P3. Created in BioRender. pang, J. (2026) https://BioRender.com/xg9rbu6.

Disturbed mitochondria–ER interactions destroy calcium homeostasis, lipid metabolism, mitochondrial dynamics, and lysosomal dysfunction, and lead to impaired mitochondrial accumulation due to abnormal mitophagy. In Treg cells, the pro-inflammatory cytokine IL-21 disrupts MAMs and upregulates glycogen synthase kinase 3 β—a negative regulator of VDAC1. This inhibition of VDAC1 perturbs pyruvate entry into mitochondria, triggering a maladaptive hypermetabolic state that reprograms Tregs into a pro-inflammatory phenotype. Notably, this IL-21-driven metabolic gene signature is enriched in intestinal Tregs from patients with CD, confirming the clinical relevance of this pathway [140]. Concurrently, in pyruvate dehydrogenase kinase 4-deficient CD4+ T cells, fewer membrane contact sites with the ER are responsible for calcium transfer between organelles, inhibiting T-cell activation and thus improving colitis in mice [141]. In UC patients, heightened inflammation and microbial signals upregulate ORMDL3. This protein accumulates at and stabilizes MAMs by interacting with Fis1. This enhanced MAMs integrity promotes mitochondrial fragmentation and dysfunction, creating a platform for the robust assembly and activation of the NLRP3 inflammasome, leading to excessive IL-1β production that drives disease progression [142]. However, research on the role of the imbalance of mitochondrial interactions with other organelles within IECs in IBD is still lacking. Moreover, aging disrupts the IP3R–MAM-mediated calcium oscillation loop. This breakdown impairs mitochondrial calcium uptake, leading to deficient autophagic flux. Consequently, accumulated cellular debris further damages mitochondrial function and MAM integrity, creating a vicious cycle of irreversible calcium decline and autophagic failure that drives stem-cell exhaustion [143] (Fig. 3a). Whether this mechanism works in ISCs under inflammation needs to be further clarified. Further studies are needed to classify whether these mechanisms function in IECs to induce IBD pathogenesis.

As described previously, mitochondrial metabolism-derived substances can undergo epigenetic modifications of cellular DNA, yet reports on their levels in IBD patients are inconsistent, varying by metabolite, disease subtype, and biological compartment (e.g. plasma, urine, feces) [111, 144, 145], and epigenetic alterations are nevertheless a recognized feature of IBD; for instance, reduced expression of the histone methyltransferase nuclear receptor binding SET domain protein 2 has been identified in the intestinal epithelium of IBD patients and mouse models [146], and DNA hypomethylation of genes in the major histocompatibility complex class I pathway has been documented in CD [147]. In addition, the levels of histone acetylation in IECs are reduced in UC patients, and inhibition of histone deacetylase can restore intestinal-barrier function and reduce intestinal inflammation [148, 149]. Beyond mere correlative findings, experimental evidence directly linking mitochondrial metabolites to epigenetic reprogramming in IBD remains scarce. While metabolites such as succinate are implicated in epigenetic regulation that exacerbates inflammation, their cellular origin is often ambiguous. A seminal study demonstrated that elevated succinate in the IBD microenvironment can enter regulatory Tregs and impair their function via a specific post-translational modification switch: it downregulates 2-oxoglutarate dehydrogenase complex, reduces succinyl-CoA, diminishes FOXP3 succinylation, and thereby triggers its ubiquitin-mediated degradation, ultimately weakening immune suppression and worsening colitis [150]. A notable exception demonstrates that, in Lgr5+ ISCs following acute gastrointestinal graft-versus-host disease, decreased succinate dehydrogenase leads to succinate accumulation. This in turn induces global DNA hypermethylation, causing persistent metabolic dysfunction and a heritable functional impairment in stem cells even after clinical resolution [151] (Fig. 3b). Taken together, the evidence summarized in this section supports the view that mitochondrial abnormalities in IECs represent more than secondary consequences of ongoing inflammation. We argue that defects in bioenergetic capacity, mitochondrial dynamics, quality control, and biogenesis can arise as “first-hit” events that exert direct effects on epithelial viability and barrier integrity. Importantly, the observation that many of these alterations occur prior to, or independently of, overt immune activation—often reinforced by genetic susceptibility loci linked to mitochondrial function—leads us to propose that the mitochondrial dysfunction of ISCs is an upstream pathogenic driver. From the authors’ perspective, this conceptual distinction is critical: it reframes the mitochondria from a passive bystander to an active determinant that sets the stage for microbial translocation and inflammatory amplification. Recognizing primary mitochondrial defects as initiating events suggests that restoring mitochondrial homeostasis may provide a preventive window to preserve barrier integrity and interrupt the transition from preclinical states to clinical IBD.

### Secondary barrier breakdown

#### Dysregulation of the mitochondria–microbiota interaction leads to intestinal biological barrier damage

IBD patients present a decreased abundance and varied composition of the gut microbiota and its metabolites [152, 153]. Alula *et al.* treated Phb1ΔPC and Phb1ΔIEC mice along with their Phb1fl/fl littermates with broad-spectrum antibiotics to investigate whether spontaneous ileitis due to mitochondrial dysfunction is influenced by the intestinal microbiota. These findings revealed that mitochondrial dysfunction in IECs or specific PCs alone does not cause intestinal inflammation [80]. Similarly, Urbauer *et al.* reported significant intestinal injury in HSP60^–/–^ mice, which was particularly influenced by the gut microbiota, with *Bacteroides* showing pronounced metabolic adaptations [154]. These studies collectively implicate gut microbes as indispensable factors for the initiation of mitochondrial dysfunction-induced IBD.

In IBD, impaired mitochondrial OXPHOS elevates luminal oxygen levels, creating a niche for the expansion of oxygen-utilizing facultative anaerobes [29, 155]. Deficiency in the mitochondrial protein MCJ increases susceptibility to colitis in mice—a phenotype driven by the enrichment of IBD-associated bacteria, such as *Ruminococcus gnavus*, *Prevotella*, and *Oscillospira*—in the host’s gut microbiota. Furthermore, these mice exhibit a specific high-IgA coating of Proteobacteria post-colitis induction, which may represent an ineffective or even detrimental host immune response [156]. In turn, some of these pathogens can directly attack and damage IEC mitochondria. For instance, infection with gram-negative bacteria such as pathogenic *Escherichia coli* LF82 disrupts mitochondrial integrity, causing swelling, cristae loss, metabolic dysfunction, and ultimately epithelial cell death [157, 158]. Similarly, the VacA toxin from *Helicobacter pylori* targets the inner mitochondrial membrane, increasing its permeability, dissipating the proton gradient, and thereby compromising OXPHOS [31]. Studies indicate that sulfate-reducing bacteria increase in IBD patients, leading to elevated levels of hydrogen sulfide, which can inhibit mitochondrial ETC functions and induce genotoxic damage to the epithelium, compromising the mucus barrier [159]. Research by Hu *et al.* revealed reduced levels of SCFA-producing bacteria in CD patients, which subsequently downregulated glucose uptake, FAO, OXPHOS, and mitochondrial biogenesis in IECs [160]. These findings delineate a pathogenic cycle in which microbiota-derived metabolites impair mitochondrial–epithelial crosstalk, exacerbating dysbiosis and barrier breakdown, as shown in Fig. 3c. Therapeutic strategies targeting microbial metabolites or mitochondrial resilience may disrupt this self-reinforcing loop in IBD.

#### Mitochondrial dysfunction in IECs contributes to intestinal physical and chemical barrier damage

Despite their distinct roles, IECs should be viewed as a cohesive organ rather than isolated units, the function of which depends on its overall intactness. Alongside the chemical barrier and the cellular immune system, the epithelial layer serves as the first line of defense. However, mitochondrial dysfunction in IECs can compromise the function of intestinal barriers, potentially leading to systemic infections and tissue damage. Thus, we hypothesize that mitochondrial dysfunction significantly contributes to the pathogenesis of IBD.

##### Adenosine triphosphate depletion and ROS lead to a reduction in tight junctions

As mentioned previously, mitochondria supply energy for tight-junction protein production, so mitochondrial dysfunction leads to a reduction in the number of tight-junction proteins. Jackson *et al.* identified ROS as disruptors of tight-junction protein interactions [161]. Chamseddine *et al.* further revealed that the unfolded protein response regulator activating transcription factor 5 is crucial for maintaining intestinal-barrier integrity during enteric infection. ATF5ΔIEC mice exhibited pathological villus/crypt remodeling and increased systemic pathogen dissemination. Uninfected mutants presented decreased tight-junction proteins (E-cadherin and ZO-1) with abnormal punctate E-cadherin distributions, indicating compromised cell adhesion, which was confirmed by elevated serum biomarkers of intestinal permeability (diamine oxidase and intestinal fatty-acid-binding protein) [162]. Knocking down the expression of a lipid transfer protein, steroidogenic acute regulatory protein-related lipid transfer domain-containing protein 7, in IECs, which helps maintain mitochondrial membrane stabilization and respiration function, also results in a reduction in the number of tight junctions [163]. In conclusion, mitochondrial dysfunction in IECs leads to a reduction in tight-junction integrity via several mechanisms to increase intestinal permeability, eventually causing inflammation.

##### Mitochondrial dysfunction leads to a decrease in the number and function of IECs

Mitochondrial dysfunction in IECs drives epithelial depletion and functional decline through multiple mechanisms. IBD patients are characterized by reduced IEC diversity and decreased ISC differentiation and migration, which negatively affects intestinal healing. Elevated expression of PGC1α in apical IECs promotes OXPHOS but fails to upregulate antioxidant defenses, and deficiency of CKMT1, a mitochondrial isozyme of creatine kinases, can lead to ROS accumulation, which triggers excessive IEC apoptosis [164, 165]. Genetic ablation of the mitochondrial chaperone HSP60 in ISCs reduces Lgr5 expression, skewing differentiation toward dysfunctional PCs and compromising organoid-forming capacity—a defect reversible by restoring mitochondrial respiration [85]. Psychosocial stress induces *Lactobacillus*-derived indole-3-acetate, which disrupts the mitochondrial function of ISCs, blocking ISC differentiation and perpetuating epithelial attrition [166]. Mitochondrial damage also impairs ISC function by disrupting the ISC niche. For example, two recent studies demonstrate this mechanism. One shows that loss of the RNA-binding protein HuR in mice disrupts mitochondrial function in PCs—specifically by downregulating PHB1—which reduces niche signals and suppresses ISCs activity, leading to epithelial atrophy [167]. Another study finds that high-dose tamoxifen directly damages enterocyte mitochondria, impairing fatty acid oxidation and inducing ER stress in PCs, thereby inhibiting ISC proliferation and differentiation and causing intestinal injury [168]. Concurrently, mitochondrial dysfunction in goblet cells impairs mucin synthesis and secretion, thinning the protective mucus layer and enabling bacterial invasion into the inner mucosa [169]. Overall, mitochondrial dysfunction can reduce the number of IECs by restricting ISC function and promoting IEC apoptosis.

Migratory bacteria promote intestinal inflammation, the most-studied of which are lipopolysaccharides, which are produced by gram-negative bacteria and can lead to IBD-like changes through the activation of toll-like receptor (TLR)-4 [170], increasing intestinal epithelial tight permeability. The inhibition of lipopolysaccharide-related pathways has been shown to ameliorate intestinal inflammation, confirming these mechanisms [171]. Overall, mitochondrial failure in IECs converges in epithelial depletion and functional decline, driving a vicious cycle of inflammation and tissue damage in IBD.

#### As the immune activator, mitochondrial signaling fuels inflammatory cycles

Research reveals a multilayered mitochondria–immune interplay that shapes gut homeostasis versus inflammation. However, few studies have elucidated the direct crosstalk between mitochondria in IECs and immune cells. Dietary cysteine supports epithelial CoA synthesis, promoting intra-epithelial CD8αβ^+^ T-cell expansion and IL-22 production, which enhances ISC-mediated repair after injury [172]. Moderate CD4^+^ T-cell-derived IFN-γ aids intestine regeneration by stimulating mitochondrial activity [173]. However, upon severe epithelial damage, released mitochondrial transcription factor A–mtDNA complexes activate dendritic cell cGAS–stimulator of interferon genes (STING)–IRF3/NF-κB signaling, driving IL-12 family cytokines and pathogenic Th1/Th17 responses [174]. Furthermore, cytotoxic T-cell attack depletes mitochondrial complex-II subunit succinate dehydrogenase subunit A in enterocytes, suppressing OXPHOS and aggravating injury [175]. Together, these pathways illustrate a continuum from protective mitochondrial–immune cooperation to a vicious cycle of mitochondrial damage and inflammatory amplification.

##### Mitochondria-derived damage-related molecular patterns act as disease amplifiers for IBD

Substances produced by damaged mitochondria can act as damage-related molecular patterns (DAMPs) to activate inflammatory responses and amplify diseases because mitochondria may originate from an endosymbiotic fusion event between *Alphaproteobacteria* and a host cell, the components of which can be recognized by pattern-recognition receptors, such as mtDNA, Cyt c, and ROS [176] (Fig. 3d). Circulating mtDNA can drive mucosal inflammation and barrier dysfunction. Studies have revealed that elevated plasma mtDNA levels in IBD patients [177] and colitis mice [178] correlate with disease activity and severity, suggesting its role as a biomarker and pathogenic mediator. Additionally, mtDNA released from IECs can also activate inflammation in other cells via extracellular vesicle transduction [179]. MtDNA release primarily stems from dysregulation of the permeability of mitochondrial membranes, particularly VDAC1-oligomerization-induced outer membrane pore formation [180]. When mtDNA is exposed to the cytoplasm, three main DNA-sensing signaling pathways are activated, including the cyclic guanosine monophosphate–adenosine monophosphate synthase (cGAS)–STING, the inflammasome pathway, and TLR9.

In the cGAS–STING pathway, mtDNA binding to cGAS triggers STING–TBK1–IRF3 signaling, which induces type I interferons [181, 182]. While STING activation acts as a key sensor of the gut microbiota in type 3 innate lymphoid cells to help maintain immune tolerance [183], it has been detected in the IECs of UC patients [184] and its inhibition can markedly attenuate DSS-induced colitis in mice [185]. Therefore, the paradoxical dual nature of cGAS–STING signaling underscores the need for further studies to understand the exact function of this signaling pathway in IBD. In the inflammasome pathway, pattern-recognition receptors such as NOD, LRR, and NLRP3 oligomerize and bind to the adaptor molecule apoptosis-associated speck-like protein containing a caspase recruitment domain or the effector molecule pro-caspase-1 in response to DAMPs. Procaspase-1 is subsequently cleaved into its mature form, which further cleaves pro-IL-1β and Gasdermin D, promoting the inflammatory process and pyroptosis [186]. In addition, by binding to TLR9, mtDNA CpG motifs can activate NF-κB signaling, as well as mitogen-activated protein kinase, IRF7, and type-I-interferon signaling [176, 187]. Furthermore, IBD patients exhibit increased TLR9+ lamina propria inflammatory cells, linking mtDNA sensing to mucosal damage amplification [188]. However, the exact function of TLR9 in IBD is still unclear. Activation of the TLR9 pathway reduces IL-6 levels in human primary IECs [189]. Notably, a TLR9 agonist imitating bacterial DNA named cobitolimod has shown efficacy in UC patients [190]. These pathways converge to disrupt epithelial integrity through pyroptosis, cytokine storms, and impaired tight-junction regeneration. However, we should not ignore the contradictory effects they have had on intestinal inflammation under specific circumstances, such as different disease stages and the intensity of inflammatory responses, and more studies are needed to clarify the exact mechanisms involved in this process in the future.

The release of mitochondrial Cyt c is a pivotal determinant of IEC fate, directing the cellular response towards either apoptosis or pyroptosis based on the magnitude of stress. This commitment step is frequently initiated by the opening of the mitochondrial permeability transition pore, which consists of cyclophilin D, adenine nucleotide translocase, and VDAC [164]. Under moderate stress, cytosolic Cyt c triggers the canonical apoptotic pathway by forming the apoptosome with APAF1 and caspase-9, which activates the executor caspase-3 [191]. Conversely, under intense stimuli such as high bile acid, massive Cyt c release coincides with high ATP efflux. This creates a unique milieu in which Cyt c and APAF1 assemble an alternative “pyroptosome,” recruiting caspase-4/11 to directly cleave gasdermin proteins and execute lytic pyroptosis [192]. This inflammatory pathway is further amplified as oxidized Cyt c can engage microsomal systems to drive a burst of ROS production, which in turn promotes more Cyt c release, forming a dangerous feed-forward loop [193]. Notably, crosstalk exists, as activated caspase-3 can also cleave gasdermin E, converting an apoptotic signal into pyroptosis [194, 195]. However, the role of cytosolic Cyt c is not exclusively pro-death. Recent evidence reveals that it can form a complex with INPP4A, enhancing a metabolic pathway that suppresses lipid peroxidation and protects against ferroptosis [196]. Furthermore, upstream regulation of mitochondrial permeability transition pore opening is crucial; the transcription factor HSF2 inhibits this pore—a mechanism analogous to the action of the UC drug cyclosporine A, highlighting a key translational checkpoint [197]. Consequently, released Cyt c itself functions as a critical DAMP. Once in the cytosol, it initiates or amplifies destructive pathways—either through the apoptosome or the pyroptosome—that ultimately contribute to intestinal-barrier disruption and inflammation. Future studies are needed to fully elucidate the context-dependent roles of Cyt c in driving intestinal inflammation, which will be essential for evaluating its potential as a therapeutic target for conditions such as IBD.

In addition to directly damaging mitochondria, a plethora of ROS can also promote IBD by amplifying the inflammatory cascade. Clinical and preclinical studies have consistently demonstrated elevated ROS levels in the intestinal mucosa of IBD patients, which are correlated with disease severity, alongside depleted antioxidants (glutathione, SOD, and vitamin C/E) in the serum and tissues. Furthermore, a multi-omics Mendelian randomization study has revealed that oxidative-stress genes play a causal role in CD, with their expression regulated by DNA methylation and host–microbiota interactions—such as MUC1 and PRKAB1 linked *Bacillus aciditolerans* and *E. coli*—thereby providing evidence for targeted therapeutic strategies against oxidative-stress-driven inflammatory pathways [198]. Therapeutic restoration of redox balance through exogenous SOD administration in murine models or phospholipid SOD infusion in UC patients attenuates inflammation and mucosal damage, validating oxidative stress as a therapeutic target [199]. Mechanistically, mitochondrial-derived ROS amplify IBD progression through several pathways, including the inflammasome pathway and NF-κB-mediated inflammation, in which ROS activate NF-κB and upregulate IL-6 and TNF-α while promoting dysbiosis, thereby fueling a self-perpetuating cycle of immune activation and epithelial injury. These pathways converge to recruit inflammatory cells, disrupt microbial balance, and propagate mucosal damage. Critically, ROS collaborate with other mitochondrial DAMPs (e.g. mtDNA, as discussed previously), creating an inflammatory milieu in which oxidative and organelle stress pathways mutually reinforce IBD progression.

Collectively, the findings discussed in this section illustrate how primary mitochondrial dysfunction in IECs propagates into a secondary cascade of barrier failure, microbial dysbiosis, and immune activation. We propose that, once epithelial energy metabolism, redox balance, and stress resilience are compromised, the intestinal-barrier transitions from a state of “intrinsic vulnerability” to one of “active inflammatory amplification.” From our perspective, secondary barrier breakdown should be conceptualized as a self-reinforcing pathological loop rather than a simple downstream consequence of inflammation. This bidirectional crosstalk—in which mitochondria-mediated epithelial damage drives dysbiosis and dysbiotic metabolites further impair mitochondrial OXPHOS—helps explain disease persistence and the high rates of relapse observed in clinical practice, even when initial inflammatory triggers are suppressed. Importantly, distinguishing secondary barrier breakdown from primary defects provides a clearer framework for overcoming the current “therapeutic ceiling” in IBD. While conventional anti-inflammatory strategies attenuate immune-driven damage, achieving durable mucosal healing likely requires the concurrent restoration of epithelial mitochondrial homeostasis. By positioning mitochondria as both the “initiator” and the “amplifier” of barrier failure, this review provides a mechanistic rationale for integrated therapies that target metabolic restoration alongside immune modulation.

## Mitochondria-targeted therapeutic strategies for IBD

Mitochondria emerge as central hubs integrating diverse IBD pathogenesis pathways, positioning patients with dysfunctional mitochondria—characterized by impaired dynamics, defective quality control, oxidative stress, and dysregulation of mitochondria–microbiota interaction—as prime candidates for mitochondrial-targeted therapies [200]. Notably, the long-term efficacy of mesalazine in UC involves mitochondrial modulation via ROS scavenging and PGC-1α activation through a PPARγ agonist [201]. Consequently, strategies aimed at restoring mitochondrial homeostasis represent a promising frontier for IBD therapy. The following sections delineate these strategies based on their primary biological targets, synthesizing insights from preclinical models and the nascent clinical evidence, as summarized in Table 2.

**Table 2:** Mitochondria-targeted therapeutic strategies for IBD.

Target type	Name	Functional mechanism	Model			Clinical trial				
			Species	Period	Outcome	Status	Time	Doses	Period	Identifier
Fission	P110	Prevent Drp-1 binding to Fis1	Mice [115]	10 days	Reduced macroscopic disease scores by 50% and extended colon length	Not available	Not available	Not available	Not available	Not available
	Mdivi-1	Drp-1 inhibitor	Human colorectal adenocarcinoma cells [202]	2 h	Suppressed inflammatory signaling, preserved mitochondrial integrity, and maintained epithelial-barrier function	Not available	Not available	Not available	Not available	Not available
Biogenesis	Rosiglitazone	PPARγ agonist	Mice [203]	21 days	Alleviated the colitis severity	Phase 2/completed	2002.9–2008.1	4 mg twice daily	12 weeks	NCT00065065
ROS scavengers	MitoTEMPO	Antioxidant	Mice [204]	7 days	Protected from DSS-induced colitis by scavenging mitochondrial ROS	Not available	Not available	Not available	Not available	Not available
	EGCG		Mice [207]	14 days	Alleviated experimental colitis	Phase 2/completed	2008.3–2014.4	400/800 mg daily	56 days	NCT00718094
	Mitoquinone		Mice [205]	Every 3 days for a total of 14 days	Enhanced the tolerance of Pck2-deficient mice to colitis	Phase 2/ recruiting	2022.5–2025.12	40 mg daily	24 weeks	NCT04276740
Mitochondrial components	Sulfide quinone oxidoreductase	Sulfide metabolism	Mice [208]	Not available	*Sqor* CKO mice show aggravated colitis with increased oxidative-stress levels	Not available	Not available	Not available	Not available	Not available
	CRIF1 (transplantation)	A nuclear transcriptional regulator and an IMM protein	Mice [209]	Not available	CRIF1 overexpression and transplantation of CRIF1-overexpressed mitochondria alleviated colitis severity	Not available	Not available	Not available	Not available	Not available
	Coenzyme Q10	A key carrier of ETC and a fat-soluble antioxidant	Mice [211]	13 days	Ameliorated colitis by inhibiting Th17 and STAT3 signaling pathways	Phase 2/completed	2018.4–2019.3	100 mg twice daily	8 weeks	IRCT200908 22002365N17
						Early phase 1/enrolling by invitation	2024.7–2026.6	Not available	2 weeks	NCT06419335
	Nicotinamide riboside (Niagen)	Restore NAD+ levels, enhance PGC1α activity and mitochondrial structure/function	Mice [125]	7 days	Reduced colitis severity, restored mitochondrial function, and increased active PGC1α levels	Not applicable/recruiting	2024.02–2026.12	12.5 mg/kg/day	6 months to 1 year	NCT05561738
Microbiota–intestine axis	Sodium butyrate	Maintain epithelial mitochondrial function	Human colonic epithelial cell lines [212]	4–24 h	Inhibited *Escherichia coli* dissemination by maintaining mitochondrial structure and function	Not applicable/completed	2013.6–2022.6	150 mg twice a day	12 weeks	NCT05456763
	Butyrate		Human colonic organoid [213]	6 h	Induced the expression of its transporter SLC16A1 and IL18	Not applicable/completed	2020.6–2022.9	900 mg daily	90 days	NCT04879914
	Succinate		Mice [214]	7 days	Restored intestinal homeostasis through enhanced barrier function and immune modulation	Phase 4/unknown status	2017.05–2018.03	Mesalazine 4 g with hydrocortisone sodium succinate 100 mg enema once daily	2/4 weeks	NCT03110198

hDrp-1 = dynamin-related protein 1, Fis1 = mitochondrial fission 1 protein, PPARγ = peroxisome proliferator-activated receptor gamma, DSS = dextran sulfate sodium, ROS = reactive oxygen species, EGCG = epigallocatechin-3-gallate, PGC1α = PPARγ coactivator 1-α, *Sqor* = *sulfide quinone oxidoreductase*, Pck2 = phosphoenolpyruvate carboxykinase 2, CKO = conditional knockout, CRIF1 = CR6-interacting factor 1, IMM = inner mitochondrial membrane, ETC = electron transport chain, Th17 = T helper 17 cells, STAT3 = signal transducer and activator of transcription 3, NAD+ = nicotinamide adenine dinucleotide, SLC16A1 = solute carrier family 16 member 1, IL-18 = interleukin 18.

### Preclinical studies: mechanistic insights and proof-of-concept

#### Strategies restoring mitochondrial dynamics, quality control, and redox homeostasis

This category encompasses interventions targeting the core pillars of mitochondrial health: balanced fission/fusion, efficient quality control, and maintained redox equilibrium. Restoring mitochondrial dynamics and quality control represents a primary strategy. Pharmacological inhibition of excessive fission via DRP1 inhibitors (e.g. P110, Mdivi-1) prevents fragmentation-induced ROS overproduction and epithelial apoptosis *in vitro* and *in vivo* [115, 202]. Rosiglitazone (a PPAR agonist) also enhances the protective effect of cold exposure on colitis [203]. These approaches highlight the therapeutic potential of directly modulating mitochondrial morphology to interrupt a key driver of epithelial damage in IBD.

Treatments involving the removal of excess mitochondria-derived ROS can also be useful. Preclinical studies have revealed that MitoTEMPO alleviates intestinal inflammation and enhances barrier function in colitis and ileitis models [204]. Moreover, mitoquinone—a mitochondria-targeted antioxidant—alleviates murine colitis by rescuing IgA-producing cells from the impairment induced by phosphoenolpyruvate carboxykinase 2 deficiency [205]. This strategy extends to nanomaterials (e.g. ultrasmall Cu2O@His nanozymes) [206] and natural compounds such as astaxanthin and epigallocatechin-3-gallate (EGCG) [207]. Together, these diverse antioxidant strategies underscore the central role of mitochondrial oxidative stress in disease propagation and validate its neutralization as a viable therapeutic axis for mitigating inflammation and barrier dysfunction.

#### Targeting specific mitochondrial components and novel paradigms

Beyond modulating global processes such as fission and redox balance, a refined therapeutic strategy involves targeting specific molecular components within the mitochondria. This approach aims for precise intervention in the pathological cascade. Preclinical research has identified several key mitochondrial proteins as high-value targets. Deficiency in the mitochondrial enzyme sulfide quinone oxidoreductase—a crucial regulator of hydrogen sulfide metabolism—drives ferroptosis and severe barrier disruption in experimental colitis, whereas its restoration confers protection [208]. Similarly, the mitochondrial inner-membrane protein CR6-interacting factor 1 plays a vital role in maintaining organelle integrity; its overexpression, or the innovative approach, significantly improves mitochondrial morphology and attenuated colitis in mice, providing a groundbreaking proof-of-concept for organelle-based therapy [209]. Among these specific targets, coenzyme Q10—an essential component of the mitochondrial ETC and an endogenous antioxidant—can improve OXPHOS, reduce ROS, inhibit the NLRP3 inflammasome, and enhance barrier integrity in IBD models [210, 211]. Nicotinamide riboside—a precursor for the essential metabolic cofactor NAD^+^—offers another strategic approach by targeting mitochondrial bioenergetics and biogenesis. It restores NAD^+^ levels, thereby enhancing the activity of PGC-1α—a master regulator of mitochondrial function—and has been shown to reduce experimental colitis severity [125]. Collectively, these targeted strategies exemplify a shift from broad metabolic modulation toward precision intervention at distinct molecular nodes within the mitochondrion, highlighting a promising trajectory for next-generation IBD therapeutics.

Emerging evidence also highlights therapeutic potential in targeting the microbiota–mitochondria–intestine axis, including harnessing commensal bacterial metabolites (e.g. butyrate-producing strains) as therapeutic targets [212], administering butyrate [213] or succinate [214], and repurposing metabolic modulators such as metformin [215]. Moreover, a recent study demonstrated that hiPSC-derived mitochondria enhance mitochondrial function and restore immune balance *in vitro*, and ameliorate disease severity, inflammation, and fibrosis in IBD mice, suggesting their translational potential for treating inflammatory disorders [216]. Taken together, these findings underscore a broadening therapeutic landscape that moves beyond conventional immunosuppression toward strategies aimed at restoring mitochondrial and metabolic homeostasis. Targeting the microbiota–mitochondria–intestine axis—whether via microbial metabolites, metabolic drugs, or even mitochondrial transplantation—represents a promising frontier for intervening in the core pathophysiology of IBD, with the potential to simultaneously improve barrier function, resolve inflammation, and promote tissue repair.

### Clinical studies: evidence of translational impact

While preclinical models have been invaluable for elucidating mechanisms, the ultimate validation of any therapeutic strategy requires evidence from human clinical trials. Currently, the landscape of mitochondria-targeted therapies for IBD is characterized by a few pioneering compounds that have entered early-phase clinical testing, while the vast majority of strategies remain confined to preclinical investigation.

The clinical evidence, though nascent, points to several promising directions. Several completed clinical trials have confirmed that mitochondria-targeted therapeutic strategies can alleviate intestinal inflammation in IBD patients, including those involving rosiglitazone (NCT00065065), EGCG (NCT00718094), coenzyme Q10 (IRCT20090822002365N17), butyrate (NCT04879914), and sodium succinate (NCT03110198). Several mitochondria-targeted clinical trials are currently in progress. For example, the MARVEL study (NCT04276740) investigates whether Mitoquinone is a beneficial drug treatment for UC. An ongoing clinical study is further evaluating CoQ10 supplementation for improving fatigue and quality of life in patients with CD (NCT06419335), highlighting its potential applicability across IBD subtypes. Furthermore, another study (NCT05561738) aims to investigate whether supplementation with Nicotinamide Riboside, which can restore NAD^+^ levels in the body, thereby enhancing the activity of PGC1α and improving mitochondrial structure and function, can alleviate symptoms in pediatric-onset UC patients. Collectively, these early-phase trials provide initial proof-of-concept that modulating mitochondrial pathways is a clinically tractable strategy, paving the way for larger, definitive studies to establish efficacy and optimize therapeutic protocols for IBD.

### Translational gap: challenges in moving from bench to bedside

Although numerous preclinical studies in mouse models have shown positive outcomes in targeting mitochondrial dysfunction for intestinal inflammation, there is currently still a lack of direct evidence from human clinical interventions. Despite their promise, translational challenges persist. First, preclinical models cannot fully replicate human IBD heterogeneity. Second, pharmacokinetic hurdles, such as the low bioavailability of promising agents such as CoQ10, limit efficacy. Third, achieving tissue- and organelle-specific drug delivery to intestinal mitochondria is difficult, risking systemic toxicity. Therefore, more efforts are needed to find smart and targeted delivery systems, advancing lead candidates through rigorous clinical trials to develop new strategies for IBD therapy that target IEC mitochondria.

## Authors’ perspective and conceptual advances of this review

While several excellent reviews have discussed mitochondrial dysfunction in the context of IBD, these discussions are often fragmented—focusing either on broad oxidative stress or primarily on immune-cell metabolism. This review advances the current understanding by proposing an integrated, epithelial-centered framework (as synthesized in Fig. 2) that positions the IEC mitochondria as a primary regulatory hub linking barrier integrity, microbial ecology, and epigenetic programming.

A key conceptual advance presented here is the distinction between primary mitochondrial dysfunction in IECs and secondary alterations driven by chronic inflammation. Rather than viewing mitochondrial abnormalities solely as downstream consequences of the inflammatory milieu, we highlight accumulating evidence suggesting that defects in mitochondrial quality control, OXPHOS, and dynamics can precede overt immune activation and serve as upstream drivers of barrier breakdown. This perspective shifts the paradigm of mitochondria from “passive victims” of inflammation to “active determinants” of disease initiation and chronicity.

Furthermore, this review provides a novel synthesis of two emerging frontiers: MAMs and mitochondrial–epigenetic coupling. We argue that the role of mitochondria extends far beyond bioenergetics; they function as critical signaling platforms in which organelle crosstalk (via MAMs) and metabolite-driven epigenetic remodeling (e.g. via succinate or α-KG) dictate the fate and long-term mucosal memory of IECs. Additionally, we integrate the “mitochondria–microbiota vicious cycle,” illustrating how mitochondrial bioenergetic failure alters luminal oxygenation, thereby driving dysbiosis, which in turn feeds back to further impair host mitochondrial function.

Finally, by organizing evidence around these mitochondria-centered mechanisms, we provide a roadmap for precision intervention. We propose that defining “mitochondrial dysfunction signatures” in IECs could help identify patient subsets who are likely to benefit from mitochondria-targeted therapies (such as mitochondria-targeted antioxidants or metabolic modulators), potentially overcoming the “therapeutic ceiling” in current IBD treatments. This review calls for a shift toward mechanism-based, personalized strategies that restore mitochondrial homeostasis as a prerequisite for durable mucosal healing.

## Conclusions

The rising incidence of IBD underscores an urgent need for a deeper understanding of the fundamental mechanisms driving these chronic disorders. Current paradigms recognize IBD as the result of complex interactions between genetic susceptibility, environmental exposures, microbial dysbiosis, and dysregulated immune responses [217]. Within this multifactorial context, mitochondrial dysfunction has emerged not merely as a byproduct of inflammation, but as a critical node influencing disease initiation, progression, and chronicity.

Accumulating evidence from both experimental models and human studies demonstrates that mitochondrial alterations in IECs serve as a hub linking oxidative stress, epithelial-barrier disruption, and metabolic–epigenetic remodeling. Furthermore, the “mitochondria–microbiota vicious cycle”—in which impaired epithelial bioenergetics drive luminal oxygenation and dysbiosis, which in turn further damages host mitochondria—represents a self-reinforcing loop that sustains mucosal inflammation. Together, these observations position mitochondria as central regulators of intestinal homeostasis.

Importantly, while mitochondrial dysfunction is clearly implicated in IBD, whether it represents a primary pathogenic driver or arises secondarily from chronic inflammation remains a pivotal and clinically relevant question. Addressing this issue is essential for defining the “point of no return” in mucosal damage and identifying early therapeutic windows. As discussed in our perspective, shifting the focus toward primary mitochondrial defects in specific cell types, such as ISCs, may provide new insights into the mechanisms of disease persistence and the failure of current treatments to achieve deep mucosal healing.

Despite significant progress, multiple challenges remain. Translational gaps persist between preclinical discoveries and effective clinical applications, partly due to disease heterogeneity and the limitations of conventional mouse models. Emerging systems, such as patient-derived intestinal organoids and organ-on-a-chip technologies, offer promising platforms to bridge this gap by enabling mechanistic studies in more physiologically relevant settings. Moreover, the long-term safety of mitochondria-targeted therapies requires careful evaluation, as the systemic modulation of mitochondrial function may interfere with global metabolic homeostasis.

Looking forward, innovative therapeutic strategies targeting mitochondrial dysfunction hold considerable promise for IBD management. These include approaches aimed at restoring mitochondrial network homeostasis through the selective modulation of fission and fusion, enhancing mitochondrial quality control, and improving epithelial energy metabolism. Advances in single-cell multi-omics integration will facilitate the identification of mitochondrial dysfunction “signatures” in distinct patient subsets, enabling true precision-medicine strategies. In parallel, repurposing established metabolic drugs and developing intestine-targeted delivery systems, such as nanocarriers or cell-based mitochondrial transfer approaches, may accelerate the clinical translation of these findings.

In conclusion, targeting mitochondrial function in IECs represents a compelling avenue for both mechanistic exploration and therapeutic innovation in IBD. Continued efforts to integrate mitochondrial biology with epithelial, microbial, and immune perspectives will be critical for breaking the “therapeutic ceiling” currently seen in IBD treatment and achieving durable, mechanism-based, and personalized mucosal healing.
